# Compositional Characterization of Different Industrial White and Red Grape Pomaces in Virginia and the Potential Valorization of the Major Components

**DOI:** 10.3390/foods8120667

**Published:** 2019-12-11

**Authors:** Qing Jin, Joshua O’Hair, Amanda C. Stewart, Sean F. O’Keefe, Andrew P. Neilson, Young-Teck Kim, Megan McGuire, Andrew Lee, Geoffrey Wilder, Haibo Huang

**Affiliations:** 1 Department of Food Science and Technology, Virginia Polytechnic Institute and State University, 1230 Washington St. SW, Blacksburg, VA 24061, USA; jin622@vt.edu (Q.J.); johair@vt.edu (J.O.); amanda.stewart@vt.edu (A.C.S.); okeefes@vt.edu (S.F.O.); andhlee@vt.edu (A.L.); geoffw7@vt.edu (G.W.); 2 Department of Food, Bioprocessing and Nutrition Sciences, North Carolina State University, 600 Laureate Way, Kannapolis, NC 28081, USA; aneilso@ncsu.edu; 3 Department of Sustainable Biomaterials, Virginia Polytechnic Institute and State University, 230 Cheatham Hall, Blacksburg, VA 24061, USA; ytkim@vt.edu; 4 White Hall Vineyards, 5282 Sugar Ridge Road, Crozet, VA 22932, USA; mmcguire@vt.edu

**Keywords:** grape pomace, chemical composition, fatty acids, polyphenols, carbohydrates, lignin

## Abstract

To better evaluate potential uses for grape pomace (GP) waste, a comprehensive chemical composition analysis of GP in Virginia was conducted. Eight commercial white and red pomace samples (cv. Viognier, Vidal Blanc, Niagara, Petit Manseng, Petit Verdot, Merlot, Cabernet Franc, and Chambourcin) obtained from different wineries in Virginia, USA were used. For extractives, GPs contained 2.89%–4.66% titratable acids, 4.32%–6.60% ash, 4.62%–12.5% lipids with linoleic acid being the predominant (59.0%–70.9%) fatty acid, 10.4–64.8 g total phenolic content (gallic acid equivalents)/kg GP, 2.09–53.3 g glucose/kg GP, 3.79–52.9 g fructose/kg GP, and trace sucrose. As for non-extractives, GPs contained 25.2%–44.5% lignin, 8.04%–12.7% glucan, 4.42%–7.05% xylan, and trace amounts of galactan, arabinan, and mannan (less than 3% in total). Potential usages of these components were further examined to provide information on better valorization of GP. Considering the valuable extractives (e.g., polyphenols and oil) and non-extractives (e.g., lignin), designing a biorefinery process aiming at fully recover and/or utilize these components is of future significance.

## 1. Introduction

Grape pomace (GP), consists of skins, seeds, and some stems. It is the major by-product of winemaking, representing over 60% of the total solid waste produced by wineries [1]. Although a small amount of GP is used for the production of grape seed oil and polyphenol-rich extracts, it is most often composted or discarded in open areas, leading to an adverse impact on the environment [1]. GP is comprised of valuable components such as residual sugars, proteins, lipids, fibers, and phenolic compounds (procyanidins, anthocyanins, and phenolic acids) [2]. These components make GP a potential renewable feedstock for the production of food ingredients, nutraceuticals, green chemicals, and biofuels [2]. 

In the past, different efforts have been attempted to introduce GP bioproducts into different utilization processes, including solvent or biological extraction to separate bioactive compounds [3], solid-state fermentation to produce enzymes [4], temperature-controlled buffer extraction to get hydrocolloids [5], and anaerobic digestion to produce biofuels and energy [6,7]. However, a major challenge for the implementation of GP into value-added processing is the high variation of GP composition, which largely determines the processing strategies for GP valorization. The compositional variation is attributed to different grape varieties, geographic locations, and wine making procedures [8,9,10,11]. For example, the composition of GP from the common grape vine (*Vitis vinifera*) obtained from eight French vineyards showed a wide range of concentrations for each component: 3%–6% (dry weight basis, DWB) of oils, 20%–46% (DWB) of structural carbohydrates, and 20%–51% (DWB) of condensed tannins [8]. For GP in the Pacific Northwest region of the USA, 1.14%–6.33% (DWB) of fat, 1.34%–77.53% (DW) of soluble sugars, 17.28%–56.31% (DWB) of dietary fiber, and 6.04%–19.89% (DWB) of condensed tannins were reported in five GP (*V. vinifera*) samples [9].

Virginia is a significant wine producing state in the eastern United States. According to the Virginia Wine Board, 7761 tonnes of wine grapes were harvested in Virginia in 2017, consisting of 74% *V. vinifera* varieties, 20% interspecific hybrid wine grape varieties, and 6% *V. labrusca* and *V. aumerensis* [12]. These grapes are used to make wine in over 260 wineries in Virginia. The production, distribution and consumption of wine in Virginia, along with wine-related agritourism, benefits many parts of the state’s economy, amounting to a total of USD five billion annually in economic impact [12]. The burgeoning wine industry in Virginia generates an increasingly significant amount of GP wastes, creating the opportunity for further utilization. However, the comprehensive compositional analysis of the GP in Virginia area has been barely studied.

In the present study, eight GP samples, four white GP and four red GP, from three wineries in Virginia were collected for a comprehensive analysis of chemical composition including moisture, lipids, fatty acids, titratable acidity, ash, soluble sugars, structural carbohydrates, lignin, and polyphenols. The comprehensive compositional analysis and detailed discussion of each component’s utilization with related processes will be helpful in the development of an integrated process for GP valorization.

## 2. Material and Methods

### 2.1. GP Sampling and Preparation

Four white GP (WGP) samples, including Vidal Blanc (interspecific hybrid cultivar, Blacksburg, VA, USA), Petit Manseng (*V. vinifera*, Crozet, VA, USA), Viognier (*V. vinifera*, Floyd, VA, USA), and Niagara (*V. labrusca*, Floyd, VA, USA), along with four red GP (RGP) samples, Chambourcin (interspecific hybrid cultivar, Blacksburg, VA, USA), Merlot (*V. vinifera*, Crozet, VA, USA), Cabernet Franc (*V. vinifera*, Crozet, VA, USA), and Petit Verdot (*V. vinifera*, Crozet, VA, USA), were collected from three wineries in Virginia, USA during 2016 and 2017. WGPs and RGPs were sampled immediately after pressing of the de-stemmed white (pre-fermentation) and red grapes (post-fermentation), respectively. All of the pomace samples were stored at −20 °C. GPs were freeze-dried and milled to a particle size of less than 0.85 mm using a hammer mill to obtain a powder for further analysis. The powder was then stored at −20 °C until the time of compositional analyses.

### 2.2. Determination of Moisture, Protein, Titratable Acidity, and Ash

Moisture content was determined by drying original GP samples (without freezing dry) at 105 °C to a constant weight [13]. Protein content was measured by the Kjeldahl nitrogen method with a nitrogen to protein conversion factor of 6.25 [14]. Titratable acidity was determined by titration with 0.1 N NaOH using tartaric acid as a standard [15]. Ash content was determined by dry ashing at 550 °C in a muffle furnace for 12 h [14].

### 2.3. Lipid Extraction and Fatty Acid Determination

Lipids were extracted from pomace powders using a Dionex Accelerated Solvent Extractor 350 (Thermo Scientific, Sunnyvale, CA, USA) following a previous procedure with modifications [16]. Briefly, GP powder (1 g) mixed with diatomaceous earth was loaded in an extraction cell (10 mL), with hexane as the solvent. Three extraction cycles were carried out using pressure and temperature settings at 1500 psi and 100 °C, respectively for 10 min. To determine lipid yield, the extract was dried under a gentle stream of nitrogen, and the mass was recorded using an analytical balance.

For the determination of fatty acid profile of the extracted lipid, fatty acid methyl esters were prepared according to the Official AOCS method Ce 1b-89 [17]. A Shimadzu GC2010 with a TQ8030 triple quad mass spectrometer operated in Q3 single quad mode (Kyoto, Japan) was used for fatty acid methyl ester identification and quantification. A Carbowax column (60 m × 0.25 mm i.d. × 0.25 μm film thickness, Phenonemex ZB-Wax Plus, Phenonemex, Torrance, CA, USA) was used for separation, and helium was used as the carrier gas with a linear flow velocity of 25 cm/s. The temperatures of the injector and detector were 250 °C and 230 °C, respectively. The oven temperature was initially 200 °C and increased at 2 °C/min until a maximum temperature of 250 °C was reached (48 min). Identification of fatty acid methyl esters was made by comparison of mass spectra using the Wiley mass spectral library and retention characteristics (equivalent chain lengths).

### 2.4. Polyphenol Extraction, Total Phenolic Content (TPC), Total Anthocyanin Content (TAC), Total Procyanidin (TPCA), and Total Flavonoid (TF) Analyses

GP polyphenols were extracted according to a previous paper with modifications [9]. GP powder was extracted by 70% acetone/28% water/2% acetic acid (*v*/*v*/*v*) with a solid to liquid ratio of 1:4 (*w*/*v*) using an ultrasonic bath for 1 h. The temperature was kept below 45 °C by exchanging the water every 15 min. After extraction, the liquid phase was separated from the solid phase by centrifugation (3234× *g*, 15 min). The solid residue extraction was repeated two more times, and the supernatant was combined and concentrated by a rotary evaporator (40 °C). The concentrate was brought to 50 mL with distilled water for the further analyses.

TPC was determined by the Folin–Ciocalteu method [18]. Appropriately diluted GP extract (0.5 mL, diluted with distilled water) was mixed with 2.5 mL Folin–Ciocalteu reagent (0.2 N) and 2 mL saturated sodium carbonate (7.5%, *w*/*v*), followed by incubation at room temperature for 2 h. The absorbance was measured at 765 nm using a Genesys^TM^ 10S UV/VIS spectrophotometer (Thermo Fisher Scientific, Madison, WI, USA) with gallic acid as the standard.

TAC was analyzed by the pH-differential method [19]. Absorbance of GP extract was measured at 520 nm and 700 nm in dilute pH 1.0 buffer (potassium chloride, 0.025 M) and pH 4.5 buffer (sodium acetate, 0.4 M) using a UV/VIS spectrophotometer (Thermo Fisher Scientific), respectively. TAC was expressed as cyaniding-3-glucoside equivalents with a molar extinction coefficient of 26,900 L cm^−1^ mol^−1^ and a molecular weight of 449.2 g/mol.

TPCA was quantified using the 4-dimethylaminocinnamaldehyde (DMAC) method [20]. Appropriately diluted GP extract (50 μL, diluted with distilled water) was mixed with 250 μL of DMAC solution (3 mL of HCl, 27 mL of ethanol, and 0.03 g DMAC), and the absorbance was then recorded at 640 nm using a Synergy^TM^ H1 microplate reader (BioTek Instruments, Inc, Winooski, VT, USA). Procyanidin B2 was used as the standard and results were expressed as procyanidin B2 equivalents.

TF of the GP extract was determined by the AlCl_3_ colorimetric method [21]. In short, diluted GP extract (1 mL, diluted with distilled water) was mixed with 5 mL of distilled water and 0.3 mL of 5% (*w*/*v*) NaNO_2_. After five minutes, 3 mL of 10% (*w*/*v*) of AlCl_3_ was added to the mixture. Following 6 min of the reaction, 2 mL of NaOH (1 M) was added to the mixture and the total volume was brought to 10 mL with distilled water. The resulting solution was mixed vigorously, and the absorbance at 510 nm was recorded using a UV/VIS spectrophotometer (Thermo Fisher Scientific). Catechin was used as the standard and results were expressed as catechin equivalents.

### 2.5. Determination of DPPH and ABTS Scavenging Abilities

The 2,2-diphenyl-1-picrylhydrazyl (DPPH) radical scavenging activity of GP extract was determined according to a previously reported method [22]. A 0.5 mL aliquot of GP extract was added to 2.5 mL DPPH radical solution (0.06 mM) and incubated for 30 min in darkness (25 °C). The absorbance was measured at 518 nm by a UV/VIS spectrophotometer (Thermo Fisher Scientific), and Trolox was used as the standard.

The 2,2’-azino-bis(3-ethylbenzothiazoline-6-sulfonic acid) (ABTS) radical scavenging activity of GP extract was determined according to a published study by Re et al. (1999) [23]. The ABTS solution was diluted with ethanol to obtain an absorbance of 0.7 units at 734 nm determined by UV/VIS spectrophotometer (Thermo Fisher Scientific). GP extract (0.2 mL) reacted with 7.6 mL of ABTS solution for 6 min in dark and the results were measured at 734 nm with Trolox as the standard.

### 2.6. Soluble Sugars Extraction and Determination

The soluble sugars of the pomace samples were extracted using 85% (*v*/*v*) ethanol with a 1:50 of solid to liquid ratio and incubated for 30 min with constant shaking in a water bath at 50 °C [24]. The extraction procedure was repeated twice, and after centrifugation (16,639× *g*, 10 min), the supernatants were combined and placed in a rotary evaporator at 50 °C to remove ethanol. The residue was re-suspended in HPLC-grade water for analyzing the concentrations of sugars (sucrose, glucose, fructose, xylose, galactose, and arabinose) using an Agilent 1200 HPLC system (Agilent Technologies, Santa Clara, CA, USA) equipped with a 1260 refractive index detector (RID). A Bio-Rad Aminex HPX-87P column (Bio-Rad Laboratories, Hercules, CA, USA) was used with HPLC-grade water as the mobile phase (0.6 mL/min) at 80 °C. The total run time was 30 min and the injection volume was 5 μL.

### 2.7. Determination of Structural Carbohydrates and Lignin

The structural carbohydrates (glucan, xylan, galactan, arabinan, and mannan) and lignin of GP samples were analyzed according to the National Renewable Energy Laboratory procedures [25]. A two-step sulfuric acid hydrolysis was conducted to break the structural carbohydrates to sugar monomers (glucose, xylose, galactose, arabinose, and mannose) which were further quantified by HPLC. The solid residue after the acid hydrolysis was then measured gravimetrically to determine the lignin content.

### 2.8. Statistical Analysis

All procedures were done in triplicate and results were expressed as mean ± standard deviation (SD). One-way analysis of variance (ANOVA) with Tukey’s test was used to confirm differences among GP samples (at 95% confidence level). Analysis was performed using SPSS software (Version 19.0, SPSS Inc., Chicago, IL, USA) and relationships between variables were assessed using Pearson’s correlation coefficient.

## 3. Results

### 3.1. Moisture, Protein, Titratable Acidity, and Ash

The GP samples from this study were byproducts from white and red winemaking processes obtained from three wineries in Virginia, USA, representing examples of GPs that are available in Virginia (Table 1). Overall, GP samples had a moisture content from 50.7% to 69.5%. The crude protein content of WGP ranged from 6.3% to 10.6%, while the crude protein content of RGP samples ranged from 11.5% to 13.0%. In contrast to WGP that is derived from pressing grapes before fermentation, RGP is sampled after alcoholic fermentation and pressing; therefore, some yeast biomass is mixed with RGP, resulting in higher protein contents. No particular pattern was found when comparing titratable acidity (2.89%–4.66%, dry weight basis, DWB) or ash (4.32%–6.60%) among WGP and RGP samples.

### 3.2. Lipids and Fatty Acids

The lipid contents of eight GPs ranged from 4.62% to 12.5% (Table 2). Twelve fatty acids were identified in GP samples and seven fatty acids contributed to less than 2% of the total fatty acids found. The remaining five major fatty acids detected in GP samples were C16:0 (palmitic acid), C18:0 (stearic acid), C18:1n-9 (oleic acid), C18:2n-6 (linoleic acid), and C18:3n-3 (α-linolenic acid). C18:2n-6 was the major fatty acid in all GP samples, ranging from 59.0% to 70.9%, followed by C18:1n-9 (ranging from 15.5% to 20.2%), and C16:0 (ranging from 7.81% to 11.6%). For both WGP and RGP samples, unsaturated fatty acids including mono-unsaturated fatty acids and poly-unsaturated fatty acids comprised a large proportion (81.5% to 89.0%) of the total fatty acids, with mono- and poly-unsaturated fatty acids varied from 16.7% to 23.5% and from 61.1% to 72.3%, respectively (Table 2). 

### 3.3. TPC, TAC, TPCA, and TF

The comparative evaluation of polyphenols from different GPs was based on four representative indices including TPC, TAC, TPCA, and TF (Table 3). TPC of WGP ranged from 11.8 to 32.1 g gallic acid equivalents/kg (DWB). As for RGP, TPC ranged from 10.4 to 64.8 g gallic acid equivalents/kg (DWB). These results were similar to those reported in other studies [9,10].

RGP had a high amount of TAC ranging from 0.22 to 3.15 g cyaniding-3-glucoside equivalents/kg (DWB), with the Petit Verdot sample having the highest TAC (13.9 g cyaniding-3-glucoside/kg of polyphenol extract powder), which was consistent with the results from a previous study (10.1 g TAC per kg of Petit Verdot polyphenol extract powder) [26]. This high TAC found in Petit Verdot could be attributed to the variation between grape cultivars and species. For example, Petit Verdot has thicker skins compared to Merlot, Cabernet Franc, and Chambourcin [27], possibly contributing to a higher TAC where anthocyanins are enriched in the skin of grapes. Other external factors, such as vinification conditions (time, temperature, enzyme treatment, and yeast), can also influence the TAC in GP [9].

TPCA indicated the amount of oligomeric and polymeric flavan-3-ols linked by C-C bonds. The values of TPCA ranged from 7.7 to 55.3 g procyanidin B2 equivalents/kg (DWB) for GP (Table 3). TF determined by the AlCl_3_ colorimetric method in this study indicated the total content of flavones, flavonols, flavanones, and flavanonols. As can be seen in Table 3, TF values ranged from 9.87 to 20.4 g catechin equivalents/kg (DWB) for WGP, and from 10.5 to 32.2 g catechin equivalents/kg (DWB) for RGP.

**Figure 1:**
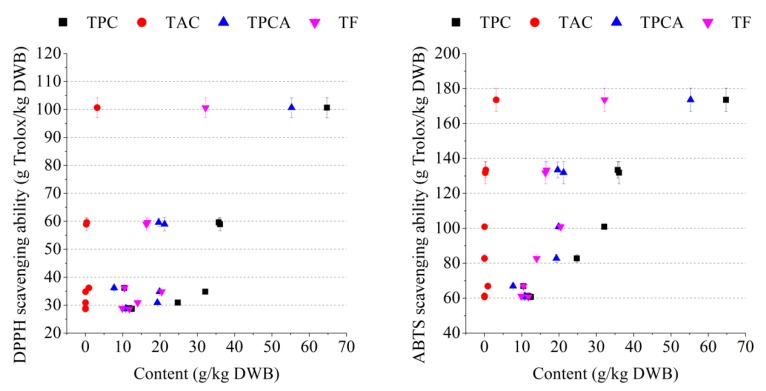
Correlation between DPPH/ABTS scavenging ability and TPC (r = 0.923/r = 0.968), DPPH/ABTS scavenging ability and TAC (r = 0.865/r = 0.679), DPPH/ABTS scavenging ability and TPCA (r = 0.911/r = 0.867), DPPH/ABTS scavenging ability and TF (r = 0.870/r = 0.876) for grape pomaces. Data were obtained in triplicate and expressed as mean ± SD.

In vitro antioxidant activities including DPPH and ABTS scavenging abilities were also tested to evaluate the functionality of GP polyphenols. In general, GP with higher TPC, TAC, TPCA, and TF contents had a higher antioxidant activity (Figure 1). According to the Pearson correlation coefficients, the antioxidant (DPPH and ABTS scavenging) activities were linearly correlated with TPC (r = 0.923 and 0.968, respectively; *p* < 0.01), TPCA (r = 0.911 and 0.867, respectively; *p* < 0.01), and TF (r = 0.870 and 0.876, respectively; *p* < 0.01). Although DPPH scavenging ability was linearly correlated with TAC (r = 0.865, *p* < 0.01), no linear correlation was found between ABTS and TAC (r = 0.679, *p* > 0.05).

### 3.4. Soluble Sugars

Of the soluble sugars identified, glucose and fructose were the major constituents, comprising over 78% of the total soluble sugars in GP (Table 4). Low levels of sucrose were detected in GP samples, corresponding to the low sucrose content in grapes and the propensity of sucrose to be hydrolyzed to glucose and fructose [28]. For RGP, the total sugar content was in the range of 7.79 to 14.3 g/kg. High amounts of soluble sugars were present in WGP (69.8 g/kg, 75.2 g/kg, and 107.6 g/kg for Petit Manseng, Viognier, and Vidal Blanc pomace, respectively).

Among WGP, the soluble sugar content of Niagara pomace sample was lower when compared to other white pomaces. The low sugar concentration could be due to several reasons based on the information provided by the winemaker who provided the GPs. First, Niagara is typically harvested at lower sugar concentrations (12 to 18 BRIX) than other varieties of grapes such as Viognier (21 to 25 BRIX) when used for winemaking. Second, the Niagara from the winery was used to make a less expensive table wine; therefore, rather than the gentle press used for Viognier, a much higher pressure was used to press Niagara to extract as much sugar as possible, leaving less sugar in pomace. Third, Niagara was mechanically harvested, and the grape skins were more severely damaged during harvesting. This allowed for more sugar and moisture to be released during the pressing process, resulting in a lower residual sugar in pomace. Finally, Niagra grapes (*V. labrusca*) are slip-skin grapes, so that the juice-containing center of the berry (pulp) physically separates more easily from the skin (the skin slides off) compared with *V. vinifera* grapes [29]. Our results herein showed that the differences in harvest and winemaking practices resulted in the different chemical compositions of GP.

### 3.5. Structural Carbohydrates and Lignin

The results for structural carbohydrates and lignin of GP are shown in Table 5. Glucan was the major structural carbohydrate in both WGP and RGP, ranging from 8.0% to 15.2% (DWB). Xylan, the main component of hemicellulose, was the second most abundant structural carbohydrate in both WGP and RGP, ranging from 4.4% to 7.1% (DWB). These results were similar to the contents of glucan (from 9.85% to 26.3%, DWB) and xylan (from 1.49% to 12.5%, DWB) in GPs from eight French vineyards [8]. Minor concentrations of galactan (0.57% to 0.93%), arabinan (0.61% to 1.03%), and mannan (0.57% to 1.55%) were also detected in both WGP and RGP samples. Lignin was the major structural component in both WGP and RGP, ranging from 25.2% to 44.5% based on a dry matter (Table 5). No apparent pattern was observed when comparing lignin content in WGP and RGP.

## 4. Discussion

The composition of GP has an important impact on its suitability for specific downstream applications and technology selection. Based on the chemical composition of GP samples detected in the present study, we summarized several value-added products which could be obtained from GP and corresponding processing technologies.

### 4.1. GP Extractives and Their Possible Uses

#### 4.1.1. Lipids

The lipids extracted in the current study are rich in unsaturated fatty acids, especially linoleic acid and oleic acid (Table 2), making them present a wide possible application in culinary, pharmaceutical, cosmetic, and medical industries [30,31,32]. However, it should be mentioned that 4.62%–12.5% of lipid contents detected in different samples were based on the dry GP which consisted of skins, seeds, and some stems, and thus the lipid content could not be directly considered as grape seed oil content. In the real practice, grape seeds will be separated from grape skins and stems at first, and lipids will be extracted from grape seeds using methods such as solvent (hexane, petroleum ether, or acetone) extraction (having high oil extraction efficiency with solvent residue) [33,34], mechanical press (providing superior quality of oil with extraction efficiency lower than 70%), and supercritical carbon dioxide extraction (high extraction efficiency without solvent residue, but with high investment and operating cost) [35]. After extraction, some purification processes including degum, deacid, decolor, and deodorization are normally guaranteed to remove unacceptable chemicals from oil/lipids before they are sent to the market [33,34].

#### 4.1.2. Polyphenols

Polyphenol is another major component that is widely recovered from GP. Based on our results, the antioxidant activity of GP extractive was mostly attributed to TPC, TPCA, and TF (Figure 1). Due to high antioxidant activity, GP polyphenolic extract can be used as synthetic antioxidant replacers and be applied in many food systems to reduce microbial spoilage, lipid oxidation, and prolong the shelf-life of food products [33]. For RGP, anthocyanins are a valuable polyphenolic compound which could be recovered and used as food grade pigments. Among the RGP samples tested in the present study, Petit Verdot pomace showed the highest TAC content (Table 3), making it a good feedstock for extraction and purification of anthocyanins. Besides being used as food ingredients, GP polyphenols can also be used in the cosmetic industry in products such as Caudalíe^®^ and Pure Super Grape^®^, for mattifying fluid or face serum production.

For the GP polyphenol extraction, the most commonly used method is solid-liquid extraction with agitation. Some advanced extraction methods such as sonicating extraction, microwave extraction, supercritical fluid extraction, and accelerated solvent extraction have also been studied to reduce extraction solvent, time, and increase the polyphenol yield [33].

#### 4.1.3. Soluble Sugars

Another extractive that can be recovered from GP is soluble sugars, especially from WGP. Although Niagara tested in the current study showed the lowest soluble sugars compared with other WGP (Table 4), in general, WGP contains relatively high amount of soluble sugars. This high concentration is mainly due to the difference in red and white wine making processes. WGP is obtained right after grape juice pressing, and RGP, on the other hand, is collected after fermenting grape pulps for several days where most sugars will be consumed by yeast cells.

The high amount of soluble sugars remaining in WGP can be recovered and purified by filtering through membrane to remove impurities, fine particles, and solid remains, and then used for the production of chemicals, such as aldonic acids, which are used in the cosmetics and plastic industry [36]. Other applications of WGP soluble sugars include substrates for aerobic or anaerobic fermentation to produce biochemicals or biofuels. A previous study showed that through yeast fermentation of WGP soluble sugars, a total of 270 L ethanol could be produced per ton of dry WGP [37]. However, due to the polyphenols that accompany the extraction of soluble sugars, the co-extracted polyphenols may inhibit the growth of some microorganisms during fermentation. The antimicrobial activity is dependent on polyphenol type, concentration, and microorganism type. For example, Gram-positive bacteria (minimal inhibitory concentration, MIC, is less than 0.2 mg/mL) are sensitive to gallotannins [38]. To reduce the negative impact of polyphenol inhibition on specific microorganism, some methods can be applied. An industrial separation method such as column chromatography could be applied to recover soluble sugars and polyphenols separately. In addition, detoxification methods such as overliming, electrochemical detoxification, and polyphenol oxidase could also be applied to remove polyphenols from soluble sugars [39,40].

### 4.2. GP Non-Extractives and Their Possible Uses

#### 4.2.1. GP Non-Extractives as Fiber-Rich Ingredients or an Energy Source

Besides GP extractives (oil, polyphenols, and soluble sugars), GP non-extractives occupied a large amount (48.2%–60.7%) of dry GP weight (Table 5). GP non-extractives can be used as dietary fiber-rich ingredients for increasing nutritional value and enhancing storability of food products. Besides food applications, GP non-extractives can also be used in thermo-chemical conversion to generate energy. A previous study found that pyrolysis of GP could generate bio-oil with a heating value of 33 MJ/kg, which is suitable for consideration as a biofuel candidate. The major problem, unfortunately, is the high acid content found in GP extract which could be corrosive to construction metals such as car engines [41]. In addition, the high potassium and calcium content in GP might cause equipment fouling in the industrial combustion [42]. Therefore, designing an extraction process at the first step to remove acids and minerals of GP could reduce the potential of ash aggregation, equipment fouling, and corrosion of construction metals in the following thermo-chemical conversion process. In the meantime, some value-added compounds such as oil, polyphenols, and soluble sugars could be recovered from GP during extraction simultaneously.

#### 4.2.2. GP Non-Extractives Separation for Individual Utilization

For the utilization of downstream bio-products, GP non-extractives can be separated into structural carbohydrates and lignin. Structural carbohydrates can be hydrolyzed to soluble sugars such as glucose and xylose, which are then used as substrates for fermentation to produce bioactive compounds, chemicals, and biofuels [37,43]. Lignin is usually discarded as waste or burnt as low-grade fuel traditionally. However, due to its attractive properties such as high carbon content, antioxidant activity, high thermal stability, and favorable stiffness, researchers have shown significant interests in converting lignin to produce multiple value-added products such as reinforcing composites, phenol-formaldehyde resins, antioxidant and antimicrobial agents, biomedical materials, carbon precursors, and smart materials [44,45]. In nature, lignin serves as a strong physical barrier by binding with structural carbohydrates tightly, therefore, finding an effective method to separate them is one of the major challenges.

The lignin content in GP is remarkably high (25.2%–44.5%, DWB, Table 5) compared with other biomass such as barley straw (13.8%, DWB), corn cobs (6.1%, DWB), corn stover (17.4%, DWB), wheat straw (17.0%, DWB), sunflower stalks (13.4%, DWB), and sugarcane bagasse (11.4%, DWB) [46], making the separation of lignin and structural carbohydrates even harder. A recent study applied dilute sulfuric acid to pretreat GP, aiming to breakdown the recalcitrant structure of GP for further enzymatic conversion but resulted in only 17% of glucose being released in the following hydrolysis [37]. Our group has evaluated NaOH pretreatments to remove lignin for the purification of structural carbohydrates in GP and achieved a maximum of 49.6% lignin removal [43]. The relatively low separation efficiency of lignin and structural carbohydrates in GP is an area of importance for future studies.

### 4.3. Limitations and Future Perspectives

One of the drawbacks in the current study is the small quantity of GP samples, since the composition of GP is highly based on cultivar, winery scale, winemaking methods, drying and storage conditions. The data presented in the current study could not represent the comprehensive characters of the studied GP cultivars. However, we have provided information of some GP cultivars available in the Virginia area and describe what valuable compounds can be recovered, how to recover them, and what processes can be used based on the GP composition. Some of the methodologies have not been fully explored and may serve as valuable information for researchers who study the utilization of GPs.

For the future perspectives, unlike most of the lignocellulosic materials, GP contains both valuable extractives (e.g., polyphenols and oil) and non-extractives (e.g., cellulose, hemicellulose, and lignin). Therefore, designing a biorefinery process to fully recover and/or utilize these components is of future significance. In addition, creating more efficient technologies and methods to increase the separation and/or recovery efficiency of valuable components is of great importance. Economic and environmental evaluation are also needed before up-grading the processes mentioned above to the industry scale.

## 5. Conclusions

The chemical composition of eight GP samples from three commercial wineries in Virginia was evaluated. In summary, lipids ranged from 4.62% to 12.5% (DWB) with linoleic acid (18:2n-6, 59.0%–70.9% of the total identified fatty acids) being the predominant fatty acid. Total polyphenols in GP showed a range of 10.4–64.8 g gallic acid equivalents/kg (DWB) with a linear correlation with in vitro antioxidant activity. The total soluble sugars were between 7.79 and 108 g/kg. As for non-extractives, lignin (25.2%–44.5%, DWB) was the major component in both WGP and RGP. Glucan (8.04%–12.7%, DWB) was the major structural carbohydrate, followed by xylan (4.42%–7.05%, DWB); and minor amounts (<3%) of other structural carbohydrates such as galactan, arabinan, and mannan were also detected. We believe that the information presented in this study by both chemical analysis and literature datamining will provide useful knowledge for more comprehensive value-added utilization of GP.

## Figures and Tables

**Table 1:** Moisture, protein, titratable acids, and ash content of white grape pomace (WGP) and red grape pomace (RGP).

Color	Cultivars	Moisture (%) ^1^	Protein (%) ^2^	Titratable Acidity (%) ^2,3^	Ash (%) ^2^
**White**	Viognier	69.5 ± 0.90 ^a^	9.51 ± 0.33 ^de^	2.89 ± 0.05 ^e^	5.07 ± 0.01 ^c^
	Vidal Blanc	67.7 ± 2.53 ^a^	6.33 ± 0.65 ^f^	4.08 ± 0.02 ^b^	4.32 ± 0.002 ^d^
	Niagara	62.0 ± 0.89 ^ab^	10.6 ± 0.59 ^cd^	4.66 ± 0.03 ^a^	5.85 ± 0.02 ^b^
	Petit Manseng	52.4 ± 0.75 ^b^	8.16 ± 0.01 ^e^	3.24 ± 0.05 ^d^	6.60 ± 0.03 ^a^
**Red**	Petit Verdot	50.7 ±4.28 ^b^	11.5 ± 0.06 ^bc^	3.16 ± 0.02 ^d^	5.36 ± 0.08 ^c^
	Merlot	50.7 ± 4.20 ^b^	12.9 ± 0.07 ^a^	3.60 ± 0.07 ^c^	6.08 ± 0.17 ^b^
	Cabernet Franc	58.1 ± 3.51 ^ab^	13.0 ± 0.21 ^a^	3.08 ± 0.02 ^de^	5.24 ± 0.10 ^c^
	Chambourcin	54.2 ± 2.97 ^b^	12.7 ± 0.12 ^ab^	3.13 ± 0.09 ^d^	4.52 ± 0.07 ^d^

Data were obtained in triplicate and expressed as mean ± SD. The superscript letters within the same row represented significant differences (*p* < 0.05). ^1^ Results were calculated based on a wet weight basis (WWB). ^2^ Results were calculated based on a dry weight basis (DWB). ^3^ Results were expressed as tartaric acid.

**Table 2:** Amounts of lipids and fatty acids composition of WGP and RGP.

	White				Red			
	Viognier	Vidal Blanc	Niagara	Petit Manseng	Petit Verdot	Merlot	Cabernet Franc	Chambourcin
**Lipid** **s (%) ^1^**	4.62 ± 0.34 ^f^	10.5 ± 0.49 ^cd^	9.54 ± 0.13 ^d^	10.9 ± 0.61 ^bc^	11.8 ± 0.40 ^ab^	11.4 ± 0.35 ^abc^	12.5 ± 0.30 ^a^	7.19 ± 0.59 ^e^
**Fatty acids (%) ^2^**								
**C12:0**	0.16 ± 0.01 ^ab^	0.11 ± 0.01 ^cd^	0.10 ± 0.003 ^d^	0.11 ± 0.005 ^cd^	0.19 ± 0.01 ^a^	0.19 ± 0.01 ^a^	0.19 ± 0.02 ^a^	0.13 ± 0.005 ^bc^
**C16:0**	11.6 ± 0.01 ^a^	9.49 ± 0.13 ^c^	9.40 ± 0.19 ^c^	8.66 ± 0.05 ^d^	9.59 ± 0.17 ^c^	9.54 ± 0.10 ^c^	10.6 ± 0.12 ^b^	7.81 ± 0.15 ^e^
**C18:0**	4.71 ± 0.06 ^b^	3.66 ± 0.06 ^e^	3.62 ± 0.05 ^e^	6.12 ± 0.09 ^a^	4.43 ± 0.04 ^c^	4.12 ± 0.04 ^d^	4.83 ± 0.07 ^b^	2.51 ± 0.03 ^f^
**C20:0**	0.78 ± 0.05 ^b^	1.08 ± 0.04 ^a^	0.71 ± 0.07 ^bc^	0.40 ± 0.03 ^e^	0.62 ± 0.04 ^cd^	0.54 ± 0.02 ^d^	0.37 ± 0.04 ^ef^	0.25 ± 0.01 ^f^
**C22:0**	0.68 ± 0.03 ^b^	0.85 ± 0.07 ^a^	0.37 ± 0.05 ^cd^	0.22 ± 0.03 ^f^	0.41 ± 0.04 ^c^	0.34 ± 0.04 ^cdf^	0.28 ± 0.03 ^dfe^	0.22 ± 0.01 ^ef^
**C24:0**	0.58 ± 0.06 ^b^	1.25 ± 0.14 ^a^	0.61 ± 0.07 ^b^	0.23 ± 0.03 ^c^	0.22 ± 0.03 ^c^	0.47 ± 0.02 ^b^	0.10 ± 0.01 ^c^	0.13 ± 0.01 ^c^
**∑ Saturated fatty acids**	18.5 ± 0.08 ^a^	16.4 ± 0.33 ^b^	14.8 ± 0.18 ^e^	15.7 ± 0.02 ^c^	15.5 ± 0.23 ^cd^	15.2 ±0.16 ^de^	16.3 ± 0.20 ^b^	11.1 ± 0.16 ^f^
**C16:1n-7**	0.41 ± 0.005 ^a^	0.25 ± 0.02 ^d^	0.38 ± 0.04 ^ab^	0.06 ± 0.01 ^e^	0.34 ± 0.02 ^bc^	0.29 ± 0.003 ^cd^	0.24 ± 0.03 ^d^	0.25 ± 0.01 ^d^
**C18:1n-9**	18.3 ± 0.05 ^c^	20.2 ± 0.19 ^b^	16.6 ± 0.20 ^de^	16.7 ± 0.14 ^d^	21.8 ± 0.42 ^a^	15.6 ± 0.11 ^ef^	16.2 ± 0.88 ^def^	15.5 ± 0.14 ^f^
**C18:1n-7**	0.82 ± 0.004 ^cd^	1.09 ± 0.09 ^a^	1.02 ± 0.01 ^ab^	0.67 ± 0.03 ^d^	1.03 ± 0.05 ^ab^	0.94 ± 0.01 ^abc^	0.90 ± 0.12 ^bc^	0.78 ± 0.04 ^cd^
**C20:1n-9**	0.18 ± 0.02 ^bcd^	0.14 ± 0.01 ^d^	0.19 ± 0.02 ^bc^	0.19 ± 0.03 ^bcd^	0.26 ± 0.01 ^a^	0.20 ± 0.02 ^b^	0.14 ± 0.01 ^cd^	0.17 ± 0.02 ^bcd^
**∑ Mono-unsaturated fatty acids**	19.7 ± 0.08 ^c^	21.7 ± 0.30 ^b^	18.1 ± 0.15 ^d^	17.6 ± 0.13 ^de^	23.5 ± 0.44 ^a^	17.1 ± 0.10 ^de^	17.5 ± 0.94 ^de^	16.7 ± 0.18 ^e^
**C18:2n-6**	59.3 ± 0.16 ^c^	59.0 ± 0.60 ^c^	64.7 ± 0.32 ^b^	65.6 ± 0.14 ^b^	59.5 ± 0.89 ^c^	66.3 ± 0.27 ^b^	64.8 ± 1.09 ^b^	70.9 ± 0.30 ^a^
**C18:3n-3**	2.53 ± 0.03 ^b^	2.85 ± 0.10 ^a^	2.34 ± 0.07 ^b^	1.05 ± 0.03 ^d^	1.62 ± 0.28 ^c^	1.47 ± 0.03 ^c^	1.34 ± 0.07 ^cd^	1.35 ± 0.02 ^cd^
**∑ Poly-unsaturated fatty acids**	61.8 ± 0.15 ^d^	61.9 ± 0.60 ^d^	67.1 ± 0.33 ^bc^	66.7 ± 0.12 ^bc^	61.1 ± 0.64 ^d^	67.8 ± 0.25 ^b^	66.2 ± 1.11 ^c^	72.3 ± 0.32 ^a^

Data were obtained in triplicate and expressed as mean ± SD. The superscript letters within the same row represented significant differences (*p* < 0.05). ^1^ Results were calculated based on a dry weight basis (DWB). ^2^ Results were calculated as a percentage of each peak area to the total identified peak area.

**Table 3:** Total phenolic content (TPC), total anthocyanins (TAC), total procyanidins (TPCA), total flavonoids (TF) of WGP and RGP.

Color	Cultivars	Content (g/kg GP)			
		TPC ^1^	TAC ^2^	TPCA ^3^	TF ^4^
**White**	Viognier	11.8 ± 0.39 ^d^	0.02 ± 0.003 ^e^	10.8 ± 0.23 ^c^	9.87 ± 0.32 ^e^
	Vidal Blanc	12.5 ± 0.56 ^d^	0.02 ± 0.003 ^e^	10.8 ± 0.29 ^c^	11.8 ± 0.92 ^e^
	Niagara	24.8 ± 0.91 ^c^	0.02 ± 0.003 ^e^	19.3 ± 1.66 ^b^	14.0 ± 0.57 ^d^
	Petit Manseng	32.1 ± 0.42 ^b^	0.04 ± 0.004 ^e^	19.9 ± 1.11 ^b^	20.4 ± 0.99 ^b^
**Red**	Petit Verdot	64.8 ± 4.12 ^a^	3.15 ± 0.07 ^a^	55.3 ± 1.15 ^a^	32.2 ± 1.16 ^a^
	Merlot	35.8 ± 1.09 ^b^	0.35 ± 0.04 ^c^	19.6 ± 1.49 ^b^	16.7 ± 0.81 ^c^
	Cabernet Franc	36.1 ± 1.08 ^b^	0.22 ± 0.006 ^d^	21.2 ± 1.19 ^b^	16.3 ± 0.81 ^c^
	Chambourcin	10.4 ± 0.79 ^d^	0.90 ± 0.05 ^b^	7.68 ± 0.31 ^d^	10.5 ± 0.13 ^e^

Data were obtained in triplicate and expressed as mean ± SD. The superscript letters within the same row represented significant differences (*p* < 0.05). ^1^ Results were expressed as gallic acid equivalents, dry weight basis (DWB). ^2^ Results were expressed as cyaniding-3-glucoside equivalents, dry weight basis (DWB). ^3^ Results were expressed as procyanidin B2 equivalents, dry weight basis (DWB). ^4^ Results were expressed as catechin equivalents, dry weight basis (DWB).

**Table 4:** Soluble sugars of WGP and RGP.

Color	Cultivars	Content (g/kg) ^1^			
		Sucrose	Glucose	Fructose	Total
**White**	Viognier	1.37 ± 0.00005 ^cd^	41.03 ± 0.03 ^b^	32.8 ± 1.31 ^c^	75.2 ± 1.34 ^b^
	Vidal Blanc	1.43 ± 0.14 ^bcd^	53.33 ± 0.09 ^a^	52.9 ± 0.50 ^a^	108 ± 0.55 ^a^
	Niagara	1.82 ± 0.06 ^ab^	6.86 ± 0.33 ^d^	7.43 ± 0.29 ^de^	16.1 ± 0.68 ^d^
	Petit Manseng	2.01 ± 0.21 ^a^	30.61 ± 1.70 ^c^	37.1 ± 2.12 ^b^	69.8 ± 0.21 ^c^
**Red**	Petit Verdot	1.15 ± 0.07 ^d^	6.18 ± 0.16 ^d^	6.95 ± 0.004 ^e^	14.3 ± 0.09 ^d^
	Merlot	1.44 ± 0.04 ^bcd^	6.05 ± 0.13 ^d^	6.49 ± 0.35 ^e^	14.0 ± 0.27 ^d^
	Cabernet Franc	1.70 ± 0.08 ^abc^	2.31 ± 0.02 ^e^	3.79 ± 0.41 ^e^	7.79 ± 0.47 ^e^
	Chambourcin	1.15 ± 0.11 ^d^	2.09 ± 0.14 ^e^	10.9 ± 0.52 ^d^	14.1 ± 0.77 ^d^

Data were obtained in triplicate and expressed as mean ± SD. The superscript letters within the same row represented significant differences (*p* < 0.05). ^1^ Results were calculated based on a dry weight basis (DWB).

**Table 5:** Glucan, xylan, galactan, arabinan, mannan, and lignin of WGP and RGP.

Color	Cultivars	Structural Carbohydrates (%) ^1^					Lignin (%) ^1^
		Glucan	Xylan	Galactan	Arabinan	Mannan	
**White**	Viognier	9.55 ± 0.19 ^c^	4.78 ± 0.24 ^c^	0.82 ± 0.08 ^ab^	0.98 ± 0.27 ^a^	0.89 ± 0.007 ^c^	33.5 ± 0.67 ^de^
	Vidal Blanc	15.2 ± 0.68 ^a^	4.62 ± 0.37 ^c^	0.93 ± 0.02 ^a^	1.03 ± 0.05 ^a^	1.23 ± 0.08 ^b^	25.2 ± 0.75 ^f^
	Niagara	12.7 ± 0.02 ^b^	5.41 ± 0.04 ^bc^	0.80 ± 0.04 ^ab^	0.77 ± 0.17 ^a^	1.26 ± 0.08 ^b^	37.6 ± 0.78 ^c^
	Petit Manseng	8.19 ± 0.16 ^d^	4.68 ± 0.25 ^c^	0.62 ± 0.06 ^b^	0.94 ± 0.33 ^a^	0.78 ± 0.03 ^c^	35.2 ± 0.48 ^cd^
**Red**	Petit Verdot	12.2 ± 0.36 ^b^	4.42 ± 0.37 ^c^	0.66 ± 0.10 ^b^	0.90 ± 0.04 ^a^	1.55 ± 0.04 ^a^	35.5 ± 1.12 ^cd^
	Merlot	11.4 ± 0.04 ^b^	4.93 ± 0.16 ^c^	0.57 ± 0.10 ^b^	0.77 ± 0.03 ^a^	1.44 ± 0.04 ^a^	30.8 ± 0.08 ^e^
	Cabernet Franc	8.72 ± 0.12 ^cd^	7.05 ± 0.30 ^a^	0.61 ± 0.05 ^b^	0.69 ± 0.10 ^a^	1.22 ± 0.003 ^b^	40.7 ± 1.29 ^b^
	Chambourcin	8.04 ± 0.42 ^d^	6.23 ± 0.13 ^ab^	0.79 ± 0.01 ^ab^	0.61 ± 0.04 ^a^	0.57 ± 0.01 ^d^	44.5 ± 0.02 ^a^

Data were obtained in triplicate and expressed as mean ± SD. The superscript letters within the same row represented significant differences (*p* < 0.05). ^1^ Results were calculated based on a dry weight basis (DWB). Limit of detections (LOD) of glucose, xylose, galactose, arabinose, and mannose were 1.12, 1.15, 1.11, 1.12, and 1.17 μg/L, respectively. Limit of quantification (LOQ) of glucose, xylose, galactose, arabinose, and mannose were 3.73, 3.83, 3.72, 3.74, and 3.9 μg/L, respectively.
